# The Forest and Tundra Nenets: differences in Y-chromosome haplogroups

**DOI:** 10.18699/vjgb-25-78

**Published:** 2025-09

**Authors:** V.N. Kharkov, L.V. Valikhova, D.S. Adamov, A.A. Zarubin, I.Yu. Khitrinskaya, V.A. Stepanov

**Affiliations:** Research Institute of Medical Genetics, Tomsk National Research Medical Center of the Russian Academy of Sciences, Tomsk, Russia; Research Institute of Medical Genetics, Tomsk National Research Medical Center of the Russian Academy of Sciences, Tomsk, Russia; Bochkov Research Centre for Medical Genetics, Moscow, Russia; Research Institute of Medical Genetics, Tomsk National Research Medical Center of the Russian Academy of Sciences, Tomsk, Russia; Research Institute of Medical Genetics, Tomsk National Research Medical Center of the Russian Academy of Sciences, Tomsk, Russia; Research Institute of Medical Genetics, Tomsk National Research Medical Center of the Russian Academy of Sciences, Tomsk, Russia

**Keywords:** gene pool, human populations, genetic diversity, Y chromosome, Nenets, генофонд, популяции человека, генетическое разнообразие, Y-хромосома, ненцы

## Abstract

The Forest and Tundra Nenets in different areas of the Yamalo-Nenets Autonomous Okrug were studied using Y-chromosome markers. The results of analyzing the genetic structure of Nenets clans using 44 STR markers of the Y chromosome are presented, taking into account their presence in subethnoses (Tundra and Forest Nenets), as well as to the Kharyuchi (“true Nenets”) and Vanuito (“foreigners”) phratries. The number of the Nenets (N = 606) includes the Tundra (N = 536) and Forest (N = 70) Nenets. Sublineage N1a2b1b1a~-B170 is specific for the clans in the Kharyuchi phratry, and sublineage N1a2b1b1b-B172, for the clans in the Vanuito phratry. Most Forest Nenets clans have haplogroup N1a2b1-B478. All males of the Pyak clan, which is prevalent in the Forest Nenets, have a specific haplogroup, N1a1a1a1a2a1c1~. The results of the study suggest that the Nenets clan associations typically have a common ancestor in the male line and are characterized by a recent founder effect. Each Nenets clan has its own specific cluster of haplotypes, equidistant from each other. The structure of Y-chromosome haplotypes and haplogroups in the Nenets gene pool includes the Nenets heritage from the Khanty and Enets. Many samples from these sample sets were shown to have rare haplotypes that were absent from the baseline data and to differ significantly from the other haplotypes found in the populations. They belong to various rare branches of the Y-chromosome haplogroups found only in these sample sets. Some samples form haplotype variants that have not been described previously and allow us to characterize the phylogeny of these lineages in more detail. The Forest and Tundra Nenets differ greatly in the composition of haplogroups, which is fully consistent with ethnological and linguistic data on the origin of these populations. The predominant haplogroups are N1a1a1a1a2a1c1~-Y13850, Y13852, Y28540 CTS9108 (xY24219, Y24375) and N1a2b1-B478, Z35080, Z35081, Z35082, Z35083, Z35084 (xB169) in the Forest Nenets, and N1a2b1b1a~-B170 (xZ35104), N1a1a1a1a2a1c~-Y13850, Y13852, Y13138, PH3340 (xY24219, Y24365) and N1a2b1b1b-B172, Z35108 in the Tundra Nenets

## Abstract (ru)

Исследованы лесные и тундровые ненцы из различных районов Ямало-Ненецкого автономного округа по маркерам Y-хромосомы. Представлены результаты генетической структуры ненецких родов по 44 STR-маркерам Y-хромосомы с учетом их принадлежности к субэтносам (тундровые и лесные ненцы), а также к фратриям Харючи («настоящие ненцы») и Вануйто («иноплеменники»). Количество ненцев N = 606 делится на тундровых (N = 536) и лесных (N = 70). Для родов, принадлежащих к фратрии Харючи, специфична сублиния N1a2b1b1a~-B170, а для родов фратрии Вануйто – N1a2b1b1b-B172. У большинства родов, относящихся к субэтносу лесных ненцев, присутствует гаплогруппа N1a2b1-B478. Все мужчины рода Пяк, доминирующего у лесных ненцев, принадлежат к специфичной гаплогруппе N1a1a1a1a2a1c1~. Результаты работы свидетельствуют о том, что родовые объединения ненцев, как правило, имеют общего предка по мужской линии, для них характерен недавний эффект основателя. Каждый ненецкий род имеет свой специфичный кластер гаплотипов, равноудаленных друг от друга. Структура гаплотипов и гаплогрупп Y-хромосомы в со ставе ненецкого генофонда демонстрирует наследие ненцев от хантов и энцев. Для многих образцов из этих выборок показаны индивидуальные редкие гаплотипы, которые отсутствуют в базах данных и значительно отличаются от других гаплотипов, обнаруженных в этих популяциях. Они относятся к различным редким ветвям гаплогрупп Y-хромосомы, обнаруженным только в этих выборках. Часть образцов формирует отдельные варианты гаплотипов, которые не были описаны ранее, и позволяет более подробно охарактеризовать филогению этих линий. Лесные и тундровые ненцы сильно различаются по составу гаплогрупп, что полностью соответствует данным этнологов и лингвистов о происхождении этих популяций. У лесных ненцев пре- обладают гаплогруппа N1a1a1a1a2a1c1~-Y13850, Y13852, Y28540 CTS9108 (xY24219,Y24375) и с небольшой частотой гаплогруппа N1a2b1-B478, Z35080, Z35081, Z35082, Z35083, Z35084 (xB169). У тундровых ненцев доминируют три гаплогруппы, N1a2b1b1a~-B170 (xZ35104), N1a1a1a1a2a1c~-Y13850, Y13852, Y13138, PH3340 (xY24219,Y24365) и N1a2b1b1b-B172, Z35108

## Introduction

The Nenets are an indigenous people of the northern territories of Western Siberia and the Eastern European part of the Urals. According to the All-Russian Census of 2021, there were 49,646 of them. They are divided into European and Siberian. European Nenets live in the Nenets Autonomous Okrug of the Arkhangelsk Region, Siberian Nenets live in the Yamalo-Nenets Autonomous Okrug of the Tyumen Region and in the Dolgano-Nenets Taimyr Municipal District of the Krasnoyarsk Territory. A very small number of Nenets live in the Khanty-Mansi Autonomous Okrug, the Murmansk Region and the Komi Republic. Together with the Enets, Nganasan and Selkup languages, the Nenets language belongs to the Samoyedic group of the Uralic language family. According to anthropological characteristics, the Nenets belong to the Mongoloids.

The Nenets are divided into Forest Nenets (living in the area of the Taz and Pur rivers of the Purovsky district of the Yamal- Nenets Autonomous Okrug) and Tundra Nenets, inhabiting the northern Priobye of the Yamal, Tazovsky and Nadym districts. The number of the Forest Nenets is very small and amounts to approximately 1,500 people. According to available data, the Forest Nenets have retained the archaic features of the Nenets community. The phenotype of the Forest Nenets has a more pronounced Mongoloid component (a less developed beard, the presence of epicanthus, a flatter face, a lower bridge of the nose), but at the same time these features are combined with Caucasian features: light eyes, a raised tip of the nose and the base of the nose. The narrow nose brings the Forest Nenets closer to the Tungus-Manchu peoples of Siberia and the Yukaghirs, typologically similar to each other; based on these data, some anthropologists suggest that it was the aboriginal tribes related to the Yukaghirs that were the predecessors of the Samoyeds in the modern territory inhabited by the Nenets (Alekseeva et al., 1972).

The Samoyedic languages are divided into two groups: Northern Samoyedic (Nenets, Enets, Nganasan) and Southern Samoyedic (Selkup and the languages of the Sayan Highlands) (Maitinskaya, 1966). Within the Nenets language, there are two dialects: the Tundra dialect, spoken by 95 % of the Nenets, and the Forest dialect. The Tundra dialect, in turn, is subdivided into three subdialects: western, eastern, and Bolshezemelsky, which formed the basis of the literary Nenets language (Tereshchenko, 1956; Khomich, 1976). The Forest dialect is used by the Nenets inhabiting the taiga zone. Understanding between Nenets who speak the Tundra and Forest dialects is very scarce, since the two dialects have significant differences in phonetics. Some phonetic features of the Tundra dialect may be related to the preservation of these sounds from the Uralic proto-language or they appeared secondarily, thanks to the language of the aborigines living in the tundra territories. The second option is supported by the fact that similar sounds are also found in the languages of the Chukchi, Koryaks, and Eskimos (UNESCO, 2010).

Such a significant difference in the two Nenets dialects in phonetic, lexical and morphological features can be explained by a closer connection of the Forest Nenets with representatives of the Enets, Nganasan, and also some features of the Forest Nenets dialect bring it closer to the Khanty language. This once again confirms that the Forest Nenets are an earlier autochthonous group of the Nenets.

The clan structure of the Nenets has been well studied by anthropologists and ethnographers (Volzhanina, 2017). Marriages among the Nenets have always been strictly exogamous. Among the Tundra Nenets, clans were united into two phratries. The first of them is Kharyuchi (“real Nenets”), which included all the clans of Samoyed origin. The second phratry is Vanuito (“foreigners”), consisting of clans that go back to the indigenous population of these territories, as well as clans of Enets and Khanty origin included in it (Khomich, 1976).

Differences in the composition and frequencies of Y-chromosome haplogroups between these two phratries are shown in our previous article (Kharkov et al., 2021). It revealed the features of haplogroup frequencies between the two Nenets phratries and the clans of the Tundra Nenets, who originated from the Samoyeds, Enets and Khanty.

The objectives of this article are to increase the samples of Tundra Nenets and compare them with new samples of Forest Nenets by Y haplogroups

The study of the structure of human population gene pools is one of the key areas of modern genetics. In recent years, there has been a real breakthrough in population genetics associated with the widespread introduction of sequencing methods, both for whole-genome genotyping of samples and for searching for new informative SNP markers in various Y-chromosome haplogroups. This makes it possible to analyze the differences in the gene pools of the Tundra and Forest Nenets of the Yamal-Nenets Autonomous Area not only at the ethnic and subethnic levels, but also at the clan level. If two people belong to different haplogroups, there can be no relationship between them in the male line. Genetic relationship is determined by the similarity of other indicators – haplotypes with an increase in the number of YSTR for a more detailed specification of the differences between them.

Until recently, the main problem was the lack of informative SNP markers for detailed analysis of the phylogenetic structure and origin of various haplogroups. New basic and terminal SNPs of the Y chromosome are extracted from the data of complete genomes for a more detailed analysis of the division of specific lines of these haplogroups. The number of SNPs discovered in recent years has already reached many thousands. Many SNPs have been confirmed on a limited set of samples and data on the frequencies of the sublineages they determine in real ethnic groups are absent or are very approximate, due to the non-representativeness of the samples studied.

Detailed analysis of haplogroups based on Y-chromosome SNP and STR genotyping is one of the most effective methods for studying the genetic diversity of human populations. It allows for a more accurate reconstruction of the origin of individual sublineages within haplogroups, calculation of their age and founder effects, as well as description of the demographic growth of populations and the phylogeny of specific variants of all Y-chromosome haplogroups. This method provides a higher level of geographic differentiation among Y-chromosome variants compared to mitochondrial DNA (mtDNA) and autosomes. These data can be used to study migration events and the history of ethnic groups (Underhill et al., 2000; Adamov, Fedorova, 2024; Adamov et al., 2024). Y-chromosome DNA markers have shown the highest level of genetic differentiation between populations compared to any other genetic systems

The aim of this study is a comprehensive analysis of haplogroup frequencies and differences in Y-chromosome haplotypes in the Forest and Tundra Nenets populations. To address the differences between them, the structure of Asian haplogroups N1a1 and N1a2 was determined and YSTR data were obtained to clarify the age and relationships between the different branches of these haplogroups.

## Materials and methods

The study material consisted of DNA samples of men from various populations of Tundra (N = 536) and Forest Nenets (N = 70). Over the past few years, we have increased the number of samples of Forest and Tundra Nenets, which included additional small clans that were not included in the population sample published in the previous article (Kharkov et al., 2021). Increasing the number of samples for various population samples of the indigenous population of Russia makes a significant contribution to the study of the specific features of their gene pools according to Y-chromosome haplogroups. Tundra Nenets were collected in the villages of Tazovsky, Antipayuta, Gyda, Samburg, Aksarka, Beloyarsk, Yar-Sale, Syunai-Sale, Nadym. Forest Nenets were collected in the villages of Tarko-Sale and Kharampur. The Kharyuchi phratry includes the Samoyedic clans Ader, Anagurichi, Vora, Vylko, Evai, Lapsui, Nenyan, Okotetto, Susoi, Serotetto, Tadibe, Taleev, Togoi, Tesida, Khudi, Heno, Yadne, Yando, Yaptunai. The Vanuito phratry includes the Samoyedic clans Vanuito, Vengo, Puiko, Yar, Yaptik, Yaungad; the Enets clans Maryik, Okovai, Ter; the Khanty clans Vekho, Nerkagi, Purungui, Salinder, Tibichi. The European Nenets clans include Laptander, Pyryrko, Syadai, Taiberi, which are not part of the Kharyuchi and Vanuito phratries. The Forest Nenets include the main clan Pyak, the Samoyedic clan Vello, Segoi; Enets clans Ayvasedo, Nyach, Ter (Kvashnin, 2011).

The material was obtained during joint scientific and practical medical expeditions from 2019 to 2024 and deposited in the bioresource collection “Biobank of the Population of Northern Eurasia”. Primary biological material (venous blood) was collected from donors in compliance with the written informed consent procedure for the study. A questionnaire was compiled for each donor with his pedigree, ethnicity and places of birth of ancestors. The study included only DNA samples from male donors who, according to the questionnaire, denied the fact of miscegenation on the paternal line with representatives of other ethnic groups in at least three generations

To study the composition and structure of Y-chromosome haplogroups, two systems of genetic markers were included in the study: diallelic loci represented by SNPs and polyallelic highly variable microsatellites (YSTRs). Using 357 SNP markers, the belonging of men to different haplogroups was determined. Some of them form the main base lines of haplogroups, while the remaining terminal SNPs are present in specific sublines in different related clans.

Genotyping of terminal SNP markers was performed using polymerase chain reaction and subsequent analysis of DNA fragments using RFLP (restriction fragment length polymorphism) analysis. For specific terminal SNPs for individual sublines, genotyping of a small number of samples was carried out based on their YSTR haplotypes and the results of NGS sequencing of the Y chromosome. The designation of haplogroups was given with reference to the ISOGG 2019 Y-DNA Haplogroup Tree.Analysis of STR haplotypes within haplogroups was performed using 44 STR markers of the non-recombining part of the Y chromosome (DYS19, 385a, 385b, 388, 389I, 389II, 390, 391, 392, 393, 426, 434, 435, 436, 437, 438, 439, 442, 444, 445, 448, 449, 456, 458, 460, 461, 481, 504, 505, 518, 525, 531, 533, 537, 552, 570, 576, 635, 643, YCAIIa, YCAIIb, GATA H4.1, Y-GATA-A10, GGAAT1B07). STR markers were genotyped using capillary electrophoresis on ABI Prism 3730 and Nanofor-05 devices

The experimental studies were carried out at the Center for Collective Use of Research Equipment “Medical Genomics” (Research Institute of Medical Genetics, Tomsk National Research Medical Center). Construction of median networks of Y-chromosome haplotypes was performed using the Network v.10.2.0.0 program (Fluxus Technology Ltd; www.fluxusengineering. com) using the Bandelt median network method (Bandelt et al., 1999). The generation age of the observed haplotype diversity in haplogroups was estimated using the ASD method (Zhivotovsky et al., 2004) based on the rootmean- square differences in the number of repeats between all markers. When calculating the age of births for individual haplogroups, single samples that significantly stood out from the general cluster of haplotypes were excluded. Calculations were performed for birth groups of at least five samples. The generation age was taken to be 30 years, the mutation rate was 0.0033 per locus per generation (Balanovsky, 2017).

The selection of derivative YSNP variants for haplogroup age estimation was performed based on the coordinate of the hg38 reference sequence, which falls within the combBED regions that roughly correspond to X-degenerated euchromatin sequences. The combBED sequence consists of 857 Y-chromosome regions with a total length of 8.47 Mb (Adamov et al., 2015). The age estimation error is calculated based on the assumption of the Poisson nature of the SNP mutation process (Poznik et al., 2013). These SNP positions were extracted from whole-genome sequencing data for 54 Nenets male samples

## Results and discussion

After genotyping of Y-chromosome SNP markers and YSTR markers, a strong difference was shown between the Forest and Tundra Nenets in the composition of haplogroups The clans of the Forest Nenets belong to two main haplogroups, N1a1a1a1a2a1c1~-Y13850, Y13852, Y28540 CTS9108 (xY24219,Y24375) and N1a2b1-B478, Z35080, Z35081, Z35082, Z35083, Z35084 (xB169), which are dominant in frequency, and only three samples belonging to haplogroup N1a2b1b1-B170 belong to the Segoi clan. The set of haplogroups is more diverse among the Tundra Nenets. The most frequent haplogroups are N1a2b1b1a~-B170 (xZ35104), N1a1a1a1a2a1c~-Y13850, Y13852, Y13138, PH3340 (xY24219,Y24365) and N1a2b1b1b-B172, Z35108, the remaining haplogroups with lower frequencies are listed in Table 1. Forest and Tundra Nenets do not coincide in the frequencies of Y-chromosome haplogroups and their clans differ greatly in the haplotypes of various sublineages.

**Table 1.**
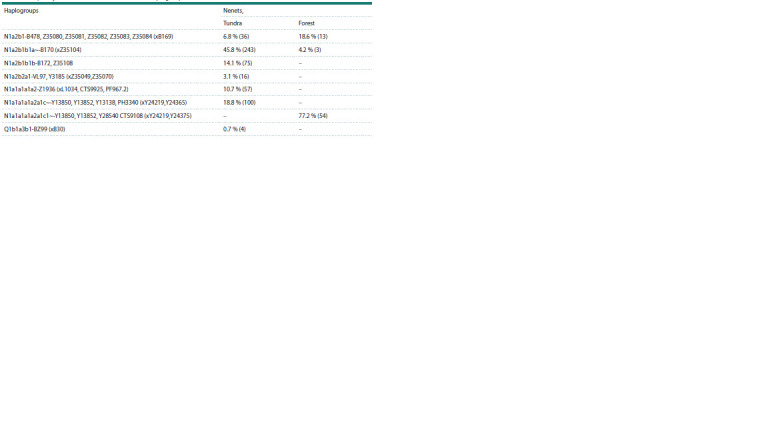
Frequency of occurrence of Y-chromosome haplogroups in Tundra and Forest Nenets

Y-chromosome haplogroup N1a2b-P43 (previously designated as N1b and N2), dominant in the Tundra Nenets (indicate the total 69.8 %), is found with uneven distribution among East Asian, Siberian and Eastern European populations. Its highest proportion is present in the populations of Western Siberia among the Nganasans (92 %), Enets (78 %) and Nenets (57 %) (Karafet et al., 2002; Tambets et al., 2004; Derenko et al., 2007; Rootsi et al., 2007; Mirabal et al., 2009; Ilumäe et al., 2016; Kharkov et al., 2021).

Two branches of the Y-chromosome haplogroup N1a1a1a1a2-Z1936 are present in the clans of the Tundra Nenets (Lapthander, Nerkagi, Salinder, Tibichi and Yar) and a parallel branch of this haplogroup is present in the Forest Nenets clan Pyak.

Haplogroup Q1b1a3b1-BZ99 was found only in three men from the Anagurichi clan of the Yamal Nenets. It has a southern Siberian origin, but a very low frequency in most Siberian populations, so it is not yet possible to compare its heritage in the Nenets with the aboriginal or alien Samoyedic population. In the whole-genome data obtained using the Admixture method, there are almost no data on the composition of Y-haplogroups of the ancient aboriginal population of these territories called Sikhirtya. According to ethnographers, the alien Samoyeds took their daughters as wives (Peoples of West Siberia, 2005). The number of men of this aboriginal population has greatly decreased due to clashes with the ancestors of the Nenets and the spread of various infectious diseases over the past several hundred years. They were hunters similar to the Yukaghirs, and their numbers did not increase from generation to generation, because they did not master reindeer herding (Khomich, 1970).

The population samples of the Gydan and Yamal Nenets completely coincide in the composition of haplogroups, but differ significantly in their frequency (Table 2). Among the Taz Nenets, the maximum frequency is characteristic of N1a2b1b1-B170, which corresponds to the proportion of men belonging to the Kharyuchi phratry. Among the Yamal Nenets, more than a third in frequency is occupied bY haplogroup N1a1a1a1a2-Z1936, to which belong almost all representatives of the Laptander clan and part of the Yar clan, who are descendants of the European Tysiya Nenets (Kvashnin, 2011).

**Table 2.**
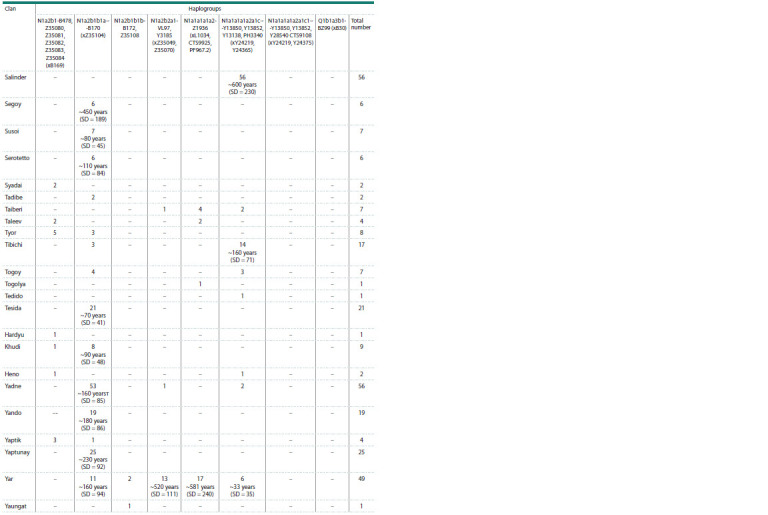
Distribution of Y-chromosome haplogroups among Tundra and Forest Nenets by clans

**Table 2end.**
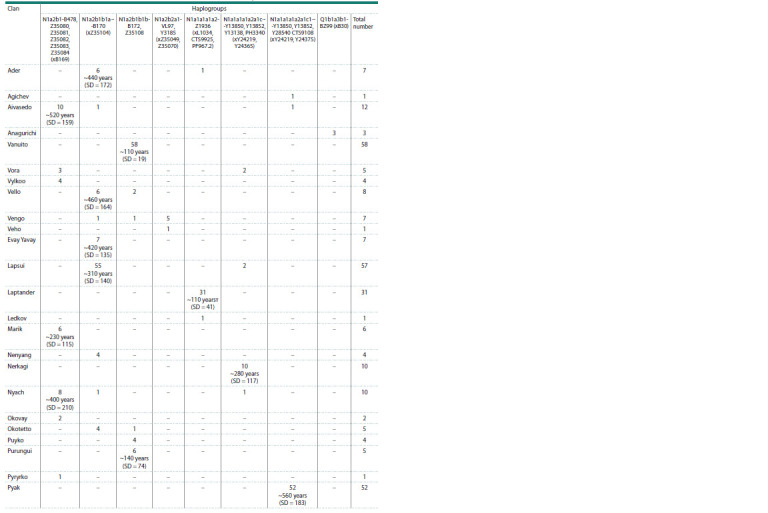
Table 2end.

**Haplogroup N1a2b1b1-B170.** This haplogroup is the most frequent among the Tundra Nenets. The Nenets are characterized by its division into specific haplotype variants, which almost completely coincide with their division into clans. It completely dominates in the Kharyuchi phratry, which includes the clans Ader, Evai (Yavai), Lapsui, Nenyan Segoi, Susoi, Serotetto, Tesida, Khudi, Yadne, Yando, Yaptunai (Table 2). Its age, estimated by us based on YSTR markers, is ~500 years (SD = 111). The age of this lineage, according to YSNP sequencing data from whole-genome data of Nenets samples, is ~1,030 years; in earlier studies, it was determined as ~1,650 years (Ilumäe et al., 2016). This haplogroup was also found in several men of the Vello clan, belonging to the Forest Nenets, which is not formally part of the Kharyuchi phratry. Another clan that is not part of this phratry, but belongs to this sublineage is Yar (part of the Vanuito phratry).

The median network N1a2b1b1a-B170, which includes men of the Kharyuchi phratry, has a typical star-shaped structure, with a clearly distinguished central haplotype, from which all the others originate. The inclusion of almost all clans of the phratry in this haplogroup confirms the theory of the community and unity of origin of the Tundra Nenets from a common male ancestor along the paternal lines. The age of this haplogroup, estimated by SNP markers (~1,030 years) does not quite coincide with the approximate time of the main migration of the Samoyedic tribes from the territory of the Kulai culture to the far North (Peoples of West Siberia, 2005). Their migration began in the 3rd-2nd centuries BC, and the common ancestor of all men of the Kharyuchi phratry along the male line was formed about a thousand years ago.

**Fig. 1.**
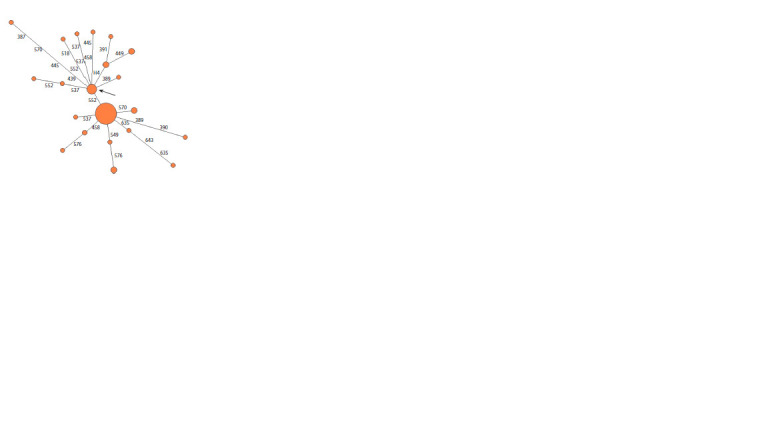
Median network of YSTR haplotypes of haplogroup N1a2b1b1a-B170 in the Lapsui clan.

**Fig. 2.**
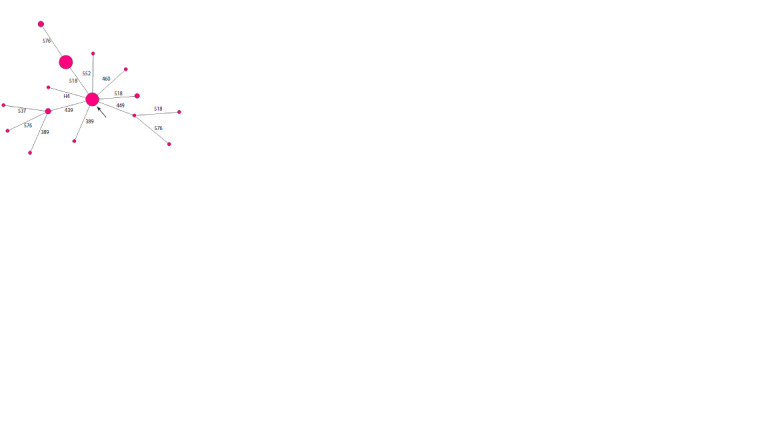
Median network of YSTR haplotypes of haplogroup N1a2b1b1a-B170 in the Yadne clan.

The age calculation of the Nenets clans that belong to this haplogroup according to YSTR is given in Table 2. The time of the common ancestor for each individual clan approximately coincides with the data of ethnographers. The Ader clan (~440 years) is one of the most ancient clans among the Nenets of the Kharyuchi phratry, and the ancestors of this clan were among the first to develop the territory to the east of Yamal. It was first recorded in the yasak book of 1695 (Kvashnin, 2001). The Lapsui clan (~310 years) separated from the third Kharyuchi wave in the mid-19th century, and the Susoi clan (~80 years), in turn, separated from the Okotetto clan in the 1920s (Kvashnin, 2001). The age of the second largest Yadne clan is ~160 years. The Figures 1 and 2 show the median networks of the clans with the largest number of men: Lapsui N = 55 (Fig. 1) and Yadne N = 53 (Fig. 2).

**Haplogroup N1a2b1b1b-B172, Z35108. **Another parallel line, N1a2b1-B172, is characteristic of the Vanuito phratry of the Tundra Nenets. Its carriers belong to the Vanuito, Vello, Purunguy, Puiko, Yar and Yaungat clans. The formation time of this more ancient Nenets branch according to YSNP is ~1,420 years. The age of these clans is also indicated in Table 2. The total age of the Purunguy, Puiko, Vello and Yar clans is ~240 years (SD = 71). Just like for the Kharyuchi phratry, the age of these clans coincides with the time of their origin. These clans were first recorded in the materials of the Surgut Church in 1880, and the Vello clan, in 1860 (Kvashnin, 2001). This lineage, present in the Nenets, was inherited by them from the Khanty ancestors in the male line. Almost each of these Nenets clans is characterized by the complete dominance of a specific subbranch N1a2b1b1b-B172, Z35108 with terminal SNPs and with a specific haplotype spectrum emphasizing the recent founder effect. In the north, the Khanty came into contact with the Nenets, some of them were assimilated by them, which is confirmed by ethnographic data, as well as our study of the clan structure of the Gydan Nenets using Y-chromosome markers (Kharkov et al., 2021).

**Fig. 3.**
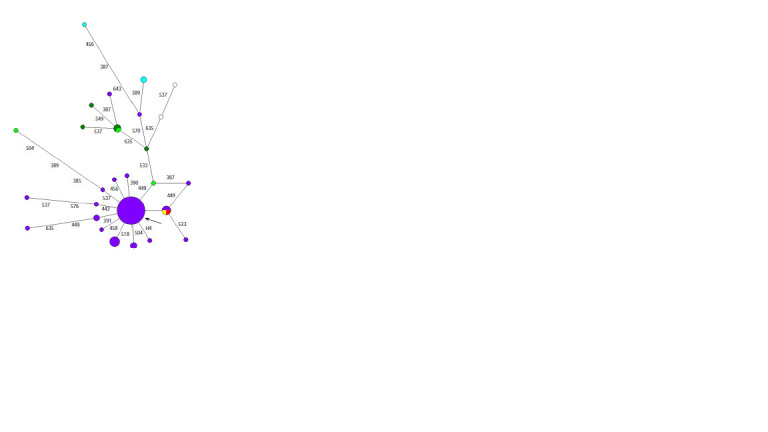
Median network of YSTR haplotypes of haplogroup N1a2b1b1a-B170 in the Vanuito, Purungui, Yar, Puiko, Wello, Okotetto and Yaungat clans The genus Vanuito is shown in lilac, the genus Purungui in dark green, the genus Yar in light green, the genus Puiko in blue, the genus Wello in white, the genus Okotetto in red, and the genus Yaungat in yellow.

The median network of this haplogroup (Fig. 3) has a clearly visible predominant haplotype, but the network itself consists of two clusters and, unlike the median network of the Kharyuchi phratry, is more fragmented. This difference between the two phratries is easily explained by the origin of these phratries. Vanuito developed on a local aboriginal substrate and also includes clans of Enets and Khanty origin, unlike Kharyuchi, who are carriers of the South Siberian Samoyed component (Prokofiev, 1940). The high frequency of this main haplotype of the median network in many men proves the strong demographic growth of these clans over the past two hundred years with a several-fold increase in the number of sons from generation to generation.

**Haplogroup N1a2b1-B478, Z35080, Z35081, Z35082, Z35083, Z35084 (xB169)**. This line is specific to the Enets clans that became part of the Nenets. It is included in the Tundra and Forest Nenets with a frequency of 6.8 % and 18.6 %, respectively (Table 1). In particular, N1a2b1-B478 was found in the Maryik, Nyach, Okovai and Ter clans – Enets clans that became part of the Tundra Nenets. This haplogroup is also present in the Forest Nenets clans Ayvasedo, Nyach and Ter, which are a branch of the Enets clan Muggadi and belong to the Vanuito phratry. The total age of haplogroup N1a2b1-B478 according to YSTR was ~680 years (SD = 202), according to YSNP data of their complete genomes, it is much older than other haplogroups of the Nenets – ~3,430 years.

**Fig. 4.**
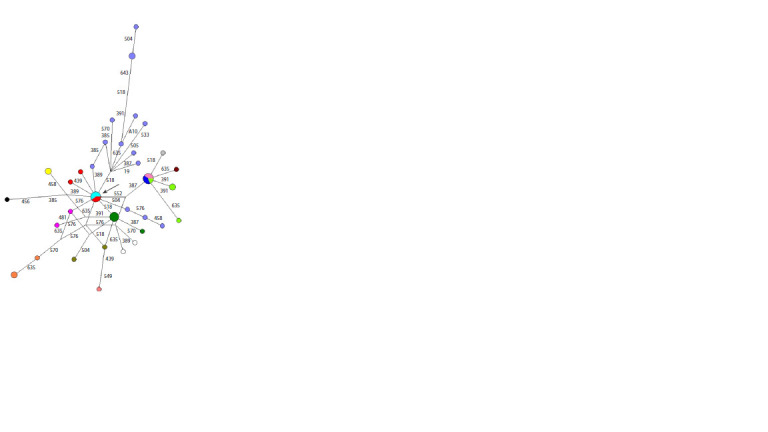
Median network of YSTR haplotypes of haplogroup N1a2b1-B478 The Aivasedo genus is shown in light blue, the Nyach genus in dark green, the Vylko genus in light green, the Syadai genus in blue, the Taleev genus in pink, the Maryik genus in light blue, the Yaptik genus in red, the Okovai genus in yellow, the Khudi genus in brown, the Hardyu genus in black, the Pyryrko genus in grey, and the Heno genus in white.

The YSTR data apparently determine the age of their separation from a common ancestor in the male line. The SNP data determine the time of occurrence of these mutations several thousand years before the demographic growth of the Samoyedic peoples in the tundra territory. In the median network constructed for these clans (Fig. 4), one can notice the absence of a dominant haplotype, as was typical for the Kharyuchi and Vanuito phratry. The demographic growth of the clans belonging to this haplogroup from generation to generation is much smaller, compared to other clans of the Kharyuchi and Vanuito phratry. Most likely, this was due to military clashes between the Enets and Nenets, the small number of descendants of the Enets clans, and their mortality from infectious diseases.

The division of this median network into small clusters may indicate heterogeneity of the origin of the Forest Nenets group. For example, the Aivasedo clan was formed by including components of the Tundra Nenets (the Chor clan) during their interaction with the Forest Enets (Vasiliev, 1973). The origin of the Nyach clan is still debated among ethnographers (Verbov, 1939; Dolgikh, 1960). According to the first version, this clan is one of the fundamental clans of the Forest Nenets of unknown origin, according to another version, it is a clan not of the Forest, but of the Tundra Nenets, which is known in the tundra under the name Nyats. All these Enets clans were assimilated by the Nenets and became part of the Vanuito phratry and the Forest Nenets. They are descendants of the local indigenous population of these territories, belonging to the Samoyedic group of languages, who mastered these areas long before the arrival of the Kharyuchi phratry, who are descendants of the later second wave of Samoyeds from Southern Siberia (Vasiliev 1975).

**Fig. 5.**
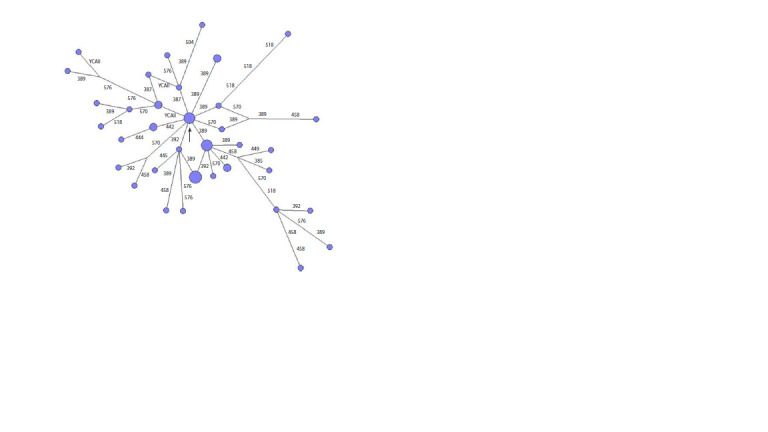
Median network of YSTR haplotypes of haplogroup N1a1a1a1a2a1c1~-Y13850 of the Pyak clan.

**Haplogroup N1a1a1a1a2a1c1~-Y13850, Y13852, Y28540 CTS9108 (xY24219,Y24375).** This haplogroup is completely dominant among the Forest Nenets, to which all men of the Pyak clan belong (Fig. 5). According to YSNP data, in comparison with the calculation of a common ancestor according to YSTR (~560 years), its age turned out to be much more ancient – ~2,680 years. There is no clearly distinguishable dominant haplotype in the median network. The Pyak clan is one of the oldest clans, first mentioned in 1627, which consists of many divisions (Vasiliev, 1973). This haplogroup was not found among the Tundra Nenets

**Haplogroup N1a1a1a1a2a1c~-Y13850, Y13852, Y13138, PH3340 (xY24219,Y24365).** This haplogroup is sister to the haplogroup of the Pyak clan. Its age according to YSTR is ~1,405 years (SD = 374). It is found in the clans of the Tundra Nenets Salinder, Tibichi, Nerkagi, Taiberi, Vora, Yar, Lar, Togoy, Lapsui.

**Haplogroup N1a2b2a1-VL97, Y3185 (xZ35049,Z35070).** This is a clade of the European branch of N1a2b, which was possibly inherited by the Forest Nenets from the Khanty or Komi. A description of this line is given in our article on the Khanty (Kharkov, 2023). It is present in the Yar and Vengo clans with a low frequency. The total age of this haplogroup in the Nenets was ~1,110 years (SD = 185).

Each of the above-mentioned clans is characterized by the presence of a dominant haplotype, as well as a pronounced genetic proximity to individuals belonging to it, which indicates a founder effect for each individual clan and a biological relationship of men of each clan along the male line. All samples belonging to different sublineages have a pronounced founder effect. They are characterized by a star-shaped phylogeny of median networks, forming separate groups by clans. Their male ancestors separated from the southern Siberian populations quite a long time ago and went north into the tundra territory.The results of genetic analysis prove that all Nenets clans are, first of all, a union of relatives on the paternal line. These data indicate a close relationship between all clans belonging to one of the phratries. With isolated exceptions, each phratry has its own specific cluster of haplotypes, equidistant from each other. Most clans of Forest and Tundra Nenets are characterized by the complete dominance of one haplogroup with a specific spectrum of haplotypes, emphasizing the recent founder effect. Two Nenets phratries differ significantly in the genetic structure of clans by Y-chromosome markers, which confirms their formation on the basis of different ancestral components from the autochthonous and migrant population. All samples belonging to different sublineages show a pronounced founder effect.

The fundamental differences between the Forest and Tundra Nenets in Y-chromosome haplogroups are shown. Three ethnic groups took part in the ethnogenesis of the Nenets: the Samoyedic group, the local aboriginal group, and clans of Khanty origin. As a result of their interaction, according to B.O. Dolgikh (1960), two phratries of the Nenets were formed. One goes back to the Samoyeds (Kharyuchi), and the other to the aborigines (Vanuito). Our results on the distribution of various Y-chromosome haplogroups in the Nenets clans are completely consistent with these data. The clan structure of the Forest and Tundra Nenets within their subpopulations was revealed.

## Conclusion

The Nenets clans of Khanty and Enets origin are completely different from each other and from the Samoyedic Kharyuchi phratry by Y-chromosome haplogroup composition. The age of YSTR haplotypes for Y-chromosome haplogroups coincides with the beginning of the demographic growth of mixed populations. The data obtained in this work supplement the information on the differences between Forest and Tundra Nenets. They are in good agreement with the accumulated body of knowledge from other disciplines studying Siberian populations: linguistics, areology, anthropology. This makes it possible to describe in more detail the history of the formation of the gene pool of Forest and Tundra Nenets. In the course of the work, an expanded set of YSTR markers was selected and used, which made it possible to move to a fundamentally new level of detail in the molecular phylogenetic structure of haplogroups and differentiation of samples

## Conflict of interest

The authors declare no conflict of interest.
